# The population frequency of predicted pathogenic variants in the genes associated with Autosomal Dominant Polycystic Liver Disease (ADPLD) and kidney cysts

**DOI:** 10.1371/journal.pone.0348109

**Published:** 2026-09-28

**Authors:** Santosh Varughese, Mary Huang, Judy Savige

**Affiliations:** 1 The University of Melbourne Department of Medicine (Melbourne Health and Northern Health), Royal Melbourne Hospital, Parkville, Victoria, Australia; 2 Department of Nephrology, Christian Medical College, Vellore, Tamil Nadu, India; Odense University Hospital, DENMARK

## Abstract

Autosomal dominant polycystic liver disease (ADPLD) commonly results from pathogenic variants in one of 7 genes (*GANAB, ALG8, ALG9, PRKCSH, SEC63* and possibly *LRP5* and *SEC61B)*. These genes are also included in kidney panels because pathogenic variants cause kidney cysts but rarely kidney failure. This study determined the population frequency of predicted pathogenic variants in the ADPLD genes in the general population. Variants for each gene were downloaded from gnomADv.2.1.1 and annotated with ANNOVAR. The population frequencies were calculated from the number of people with ‘predicted pathogenic’ variants in gnomAD: loss-of-function structural and copy number; null; and rare, computationally-damaging missense changes that affected a conserved residue. Frequencies were also estimated from the number of gnomADv.4.1 variants assessed as Pathogenic or Likely pathogenic in ClinVar. Predicted pathogenic variants affected one in 91 people using our strategy and gnomAD v.2.1.1, and one in 130 with ClinVar assessments of gnomAD v.4.1 variants. *LRP5* and *ALG8* which are associated with a milder clinical phenotype, were the commonest affected genes with both strategies. Predicted pathogenic variants in ADPLD appear more frequent in admixed American (one in 91), Finnish (one in 110) and African/African American (one in 43) people (p all < 0.0001 compared with Europeans (one in 187). Predicted pathogenic variants for ADPLD may be even more common with the finding of additional causative genes. Assessments will become more accurate too. However not all ADPLD variants result in liver cysts, nor indeed cystic kidneys, because of incomplete penetrance and variable expressivity.

## Introduction

Autosomal Dominant Polycystic Liver Disease (ADPLD) overlaps genetically and clinically with Autosomal Dominant Polycystic Kidney Disease (ADPKD) [1]. Multiple liver cysts occur in 80% of people with ADPKD due to *PKD1* and *PKD2* variants [2], especially women [3], but the cysts are often smaller and less numerous than those in ADPLD [4]. Liver cysts are also associated with autosomal recessive (AR) PKD caused by *PKHD1* [5] and *DZIP1L* [6] changes.

ADPLD results from pathogenic variants in *PRKCSH*, *SEC63*, *ALG8*, *ALG9,* *GANAB* and possibly *LRP5* and *SEC61B*[7–9], which together account for at least 40% of people with ADPLD (Table 1) [8]. We have previously reported the population frequency of *ALG9* [10]. These genes typically encode proteins involved in the transport of fluid or epithelial cell growth in the liver [11]. Some affect the glycosylation, folding or trafficking of ER proteins that process the polycystin 1 (PKD1) molecule that is affected in ADPKD [12,13].

**Table 1:** Genes, mechanisms, and clinical features in AD polycystic liver disease.

Gene name	Gene and protein features	Disease mechanism
***GANAB*** **Glucosidase II Alpha Subunit** **(MIM 104160), AD**	944 amino acids, both protein truncating and missense variants located throughout the gene in Simple ClinVar	Required for polycystin-1 and polycystin-2 maturation and localization to the cell surface and cilia
***ALG8* Asparagine-Linked Glycosylation 8 Homolog** **(MIM: 608103), AD**	526 amino acids, both protein truncating and missense variants located throughout the gene in Simple ClinVar	Required for polycystin-1 maturation and localization to the plasma membrane of the primary cilia
***ALG9* Asparagine-linked Glycosylation 9 Homolog (MIM:606941), AD**	611 amino acids, both protein truncating and missense variants located throughout the gene in Simple ClinVar	Encodes alpha-1,2-mannosyltransferase that is important in lipid-linked oligosaccharide assembly
LRP5* **LDL Receptor Related Protein 5** **(MIM: 603506), AD**	1,615 amino acids, both protein truncating and missense variants located throughout the gene in Simple ClinVar	Encodes a transmembrane low-density lipoprotein receptor that binds and internalizes ligands in the process of receptor-mediated endocytosis
***PRKCSH*** **Protein Kinase C Substrate Heavy Chain Beta Subunit Of Glucosidase II** **(MIM: 177060), AD**	528 amino acids, protein truncating variants located throughout the gene in Simple ClinVar	Required for efficient Polycystin-1 biogenesis and trafficking to the plasma membrane of the primary cilia
*SEC61B* * **SEC61 Translocon Subunit Beta** **(MIM: 609214), AD**	96 amino acids	Encodes the b-subunit protein of the Sec61 complex which is the central component of the protein translocation apparatus of the endoplasmic reticulum membrane
***SEC63*** **SEC63 Homolog, Protein Translocation Regulator** **(MIM: 608648), AD**	760 amino acids, both protein truncating and missense variants located throughout the gene in Simple ClinVar	Mediates co-and post-translational transport of certain precursor polypeptides across endoplasmic reticulum

**Based on OMIM (**https://www.omim.org/**) Genes reported in KDIGO 2025 Clinical Practice Guidelines for the Evaluation, Management and Treatment of AD polycystic kidney disease [**9**].** *However  the evidence for two genes (*LRP*, *SEC61B*) is assessed as  ‘limited’ as a cause of ADPLD by the ClinGen Gene Disease Framework.

*PRKCSH* variants are often associated with isolated PLD [14], and, in general, *PRKCSH*, *SEC63*, and *GANAB* result in a more severe phenotype than *ALG8* and *LRP5*. All these genes are also associated with multiple kidney cysts, and *ALG8* and *GANAB* variants have previously been reported to be the commonest ADPLD-related causes of kidney cysts [7]. However the ADPLD-associated genes rarely result in kidney failure [1].

These 7 genes are all associated with AD inheritance, and, while, for some genes, few variants have been reported, currently nonsense, splice site, and missense changes as well as insertions and deletions, have been described throughout most genes without obvious hotspots [11] (Fig 1, Simple ClinVar, https://simple-clinvar.broadinstitute.org/). Penetrance is more variable than for ADPKD, even within a family, and there appear to be no obvious genotype–phenotype correlations [15,16].

**Fig 1:**
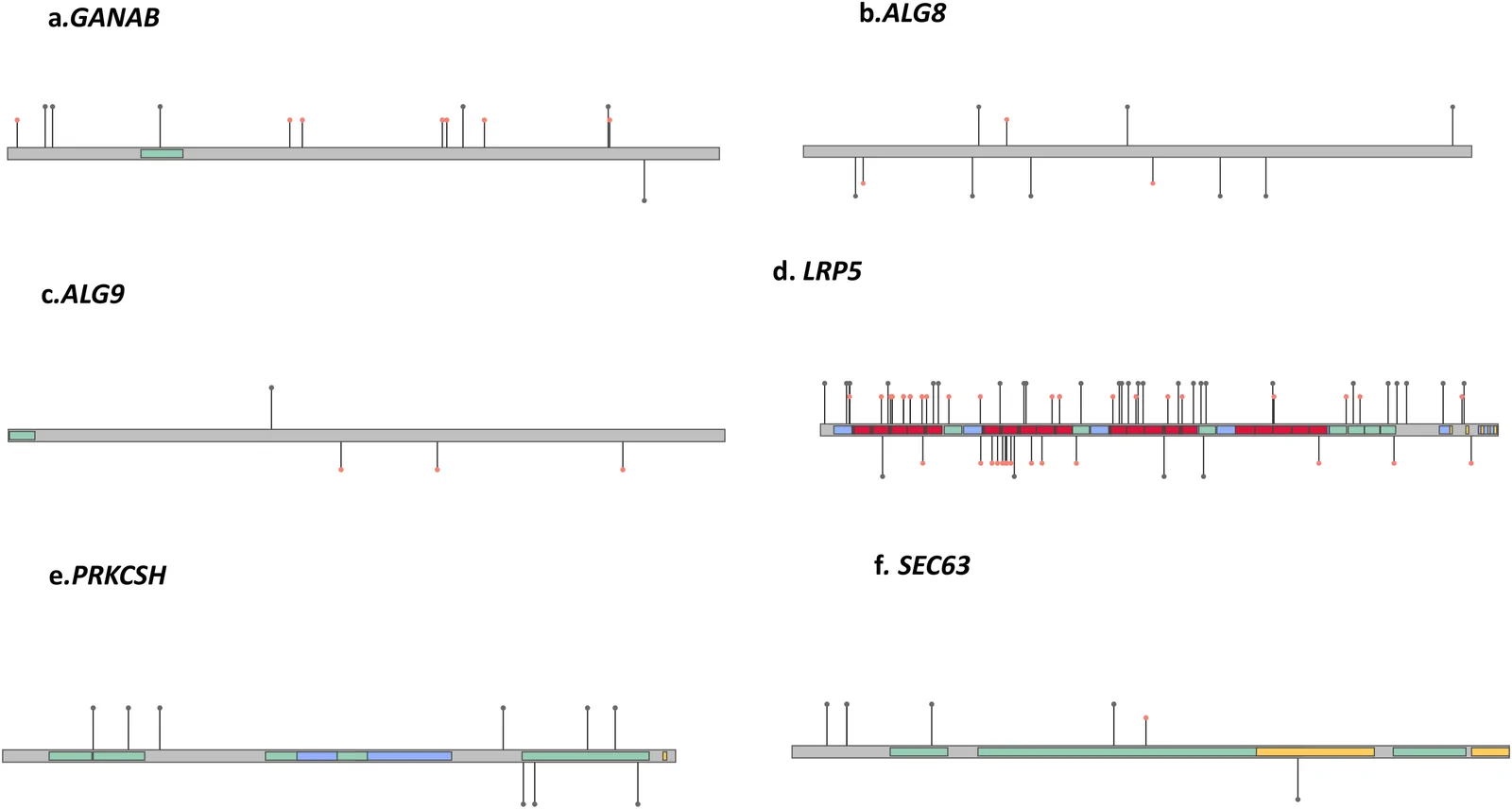
Pathogenic and Likely pathogenic variants in ADPLD from Simple ClinVar fora.*GANAB*, b.*ALG8*, c. ALG9, d.*LRP5*, e.*PRKCSH* and f.*SEC63.* There were no Pathogenic or Likely pathogenic variants for *SEC61B* in Simple ClinVar (https://simple-clinvar.broadinstitute.org/).

The reported population frequency of ADPLD varies depending on whether the diagnosis has been based on clinical features, imaging, genetics or from the association with AD or AR polycystic kidney disease. The formal diagnosis of ADPLD depends on the demonstration of at least 4 liver cysts and another affected family member, or ten cysts without a family history [17]. Population studies usually underestimate the disease burden because most polycystic liver disease is asymptomatic. Isolated polycystic liver disease has been reported in one in 100,000 people, and in fewer than one in 10,000 people using medical records and imaging reports from the Mayo clinic [18]. In one study, ADPLD affected one in 400 or one in 20,000 people when associated with ADPKD or ARPKD [5]. However the population frequency was one in 496 when truncating variants were counted in three genes (*PRKCSH, SEC63, GANAB)* from two large public sequencing databases (gnomAD v.2.0.2, BRAVO) [19].

A more accurate population frequency for ADPLD will increase clinician awareness of the condition and its association with ADPKD, improve genotype-phenotype correlations and encourage pharmaceutical companies to develop targeted treatments or at least monitor the response of liver cysts to therapies for cystic kidney disease.

The present study determined the population frequency of predicted pathogenic variants in seven genes affected in ADPLD from two minimally-overlapping population datasets (gnomAD v.2.1.1 and v.4.1). Our strategy used computational tools based on the American College of Medical Genetics and Genomics (ACMG)/Association for Molecular Pathology (AMP) criteria for pathogenicity [20]. The population frequency was calculated from the number of people with structural or copy number changes that were assessed as loss of function; together with null variants; and missense changes that were rare, structurally or functionally damaging in computational tools and affected a conserved amino acid, in gnomADv.2.1.1. This population frequency was then compared with those derived from variants found in gnomAD that had also been assessed disease-causing in the ClinVar (https://www.ncbi.nlm.nih.gov/clinvar/), LOVD (https://www.lovd.nl/) or HGMD (https://www.hgmd.cf.ac.uk/ac/index.php) databases. These results were then confirmed using ClinVar analysis of the larger gnomAD v.4.1 database.

Our approach for calculating the population frequency for ADPLD has been used for many other monogenic diseases including Alport syndrome, AD Polycystic kidney disease, Wilson disease, Menkes disease, Gitelman syndrome, Mucopolysaccharidoses, Fabry disease, and Lysosomal acid lipase deficiency [21–27], and, in some cases, these results have been confirmed independently with epidemiological, histological or biochemical studies [22,24].

## Methods

### Population datasets

Two datasets were examined: gnomADv.2.1.1 (GRCh37/hg19) and gnomAD v.4.1 (GRCh38/hg38). gnomADv.2.1.1 comprises anonymised Whole Exome Sequencing (WES, n = 141,456 samples) and Whole Genome Sequencing (WGS, n = 10,847 samples) from multiple large-scale sequencing projects of adults with diabetes, neuropsychiatric or cardiac disease and has equal numbers of men and women, together with their ancestries but not their clinical data. gnomAD v.2.1.1. also includes a Control subset (n = 60,146) of people who were age- and gender-matched for those with these diseases but were themselves unaffected.

gnomAD v.4.1 includes a larger number of samples (n = 807,162) with some examined for structural (n = 63,046) and copy number (n = 464,297) variants and with people of more diverse ancestries. gnomADv.4.1 comprises data from gnomAD v.2.1.1 (n = 314,392), mainly community-based people recruited to the UK Biobank (n = 492,770), and other sources. GnomAD v.2.1.1 and gnomAD v.4.1 variants overlap by about 20% of variants.

Variants were downloaded between August 2024 and December 2024, and reviewed in May 2025.

### IRB approval

This study did not use human participants but instead examined anonymised genomic data from gnomAD. All gnomAD participants had previously consented for the use of their anonymized demographic and genomic data so that secondary Institutional Review Board approval was not required.

### Population frequency of predicted pathogenic variants in the ADPLD genes using gnomAD v.2.1.1

The population frequency of ADPLD was calculated from variants in 7 genes (*GANAB*, *ALG8,* ALG9, *LRP5, PRKCSH, SEC61B, SEC63)* associated with ADPLD (https://curation.clinicalgenome.org/) [28]. These genes were chosen on the basis of the KDIGO guidelines [9] and a previous study [25]. Pathogenic variants in *ALG9* are associated with both AD polycystic kidney and liver disease and we have published its population frequency previously. The genes were downloaded, annotated with ANNOVAR (https://annovar.openbioinformatics.org/), and assessed for pathogenicity as we have described [26,29].

Variants in the intronic and 5’ or 3’ UTR or that were synonymous were excluded. The remaining variants were filtered using the following strategy (Fig 2).

**Fig 2:**
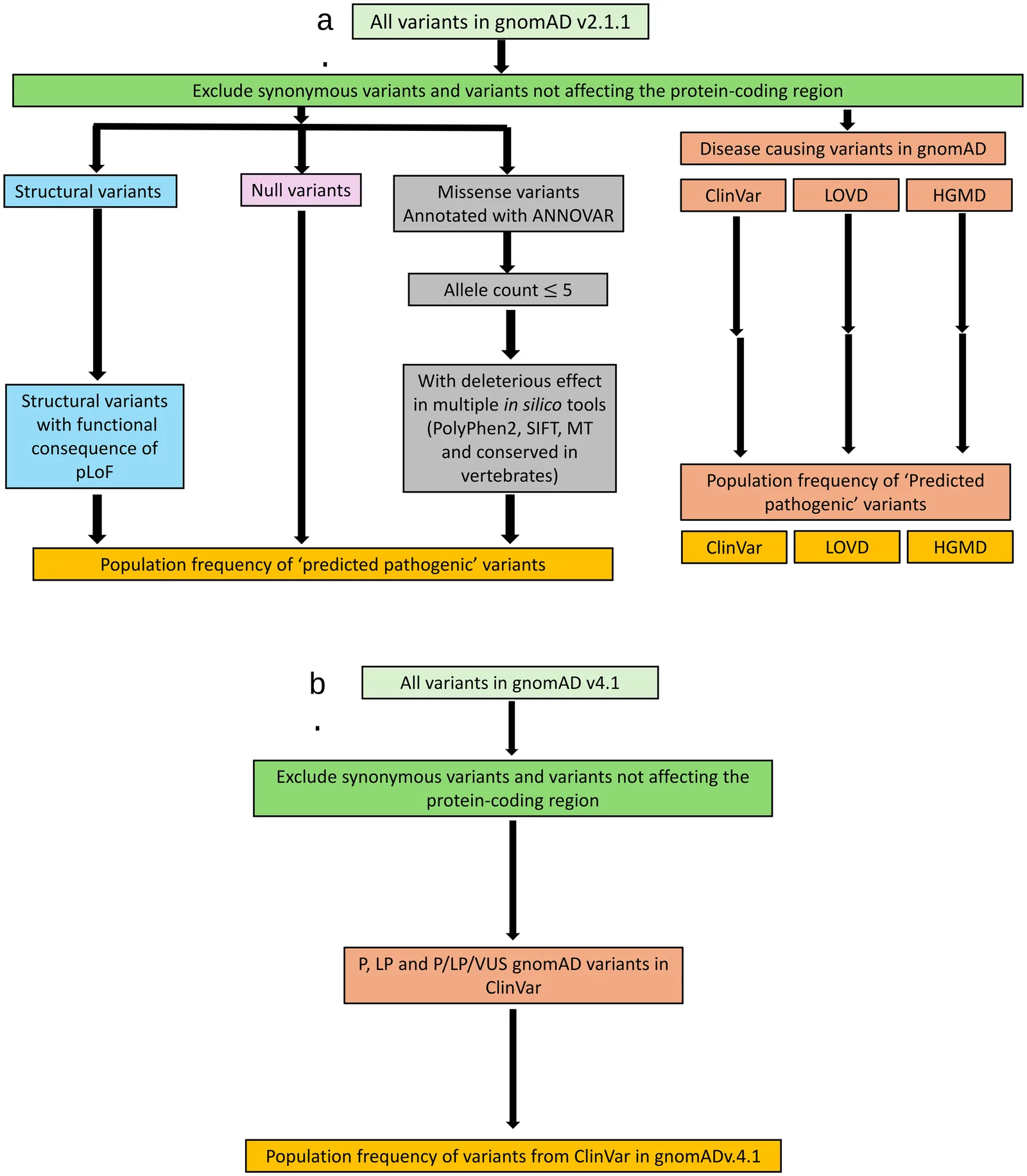
Our filtering strategy for identifying predicted pathogenic variants in the genes for AD Polycystic Liver Disease in gnomAD v.2.1.1 and v.4.1.

#### Structural variants.

Structural variants that were classified as loss of function by gnomAD and affected an exon were assessed as predicted pathogenic. The number of people with a predicted pathogenic variant in the smaller structural variant sample was corrected to be equivalent to the whole cohort. All predicted pathogenic variants were included in the total regardless of their allele frequency for structural, copy number and null variants. Structural variants were available for gnomAD v.2.1.1 and v.4.1.

#### Copy number variants.

Copy number variants that resulted in the deletion of a least one exon were assessed as predicted pathogenic. Again, the number of people with each predicted pathogenic variant was corrected for the smaller copy number sample size. Copy number variants were only available for gnomAD v.4.1.

#### Null variants.

Null variants, including terminating codons, splicing and frameshift changes (except those in the last exon or the last 50 nucleotides of the penultimate exon, which do not undergo nonsense-mediated decay), were assessed as predicted pathogenic. The canonical mRNA transcripts and the locations of the final nucleotide beyond which variants do not result in nonsense mediated decay were obtained from Alamut (https://www.sophiagenetics.com).

#### Missense variants.

Missense variants were classified as predicted pathogenic if they were rare (≤ 5) and disease-causing in the following three bioinformatic tools: PP2 (Polymorphism Phenotyping v2, PolyPhen-2) score ≥ 0.95 (http://genetics.bwh.harvard.edu/pph2/); SIFT4G (Sorting Intolerant From Tolerant) score ≤ 0.05 (https://sift.bii.a-star.edu.sg/; and MT (MutationTaster) where they were “disease causing, or probably deleterious” (D) or “disease-causing automatic and known to be deleterious” (A) (https://www.mutationtaster.org/info/documentation.html). Predicted pathogenic variants also affected an amino acid that was conserved in vertebrates (the same (*) or similar (:) in chicken, mice, humans, using Clustal Omega (https://www.ebi.ac.uk/Tools/) and the Ensembl reference sequences (http://asia.ensembl.org/index.html).

We chose to examine missense variants using multiple computational tools (PP2, SIFT, Mutation Taster, GERP) rather than metapredictors such as REVEL and BAYESdel, which both include these three tools and others. Metapredictors have the weakness that many of their tools measure the same variant feature and the performance of the better ones is diminished by other under-performers. We did not exclude variants that were homozygous in gnomAD (which is a common strategy to exclude benign changes) because our studies have found that homozygous pathogenic variants were unlikely to be included with a minor allele frequency of ≤ 5.

The accuracy of our missense variant assessment was evaluated as follows. Twenty Benign and 20 Pathogenic missense variants were selected randomly in genes for ADPLD from ClinVar (Suppl Table S1 in S1 Data), assessed by our criteria for missense changes, and the sensitivity and specificity, positive and negative predictive values calculated. Benign variants included 4 *GANAB*, 4 *ALG8*, 1 *LRP5*, 7 *PRKSH* and 4 *SEC63* missense changes; and Pathogenic variants included 4 *GANAB*, 3 *ALG8*, 8 *LRP5*, and 5 *SEC63* variants.

#### Calculation of the population frequency of predicted pathogenic variants in ADPLD genes.

The population frequencies of predicted pathogenic variants for individual genes were calculated from the total number of people with pathogenic variants in each gene divided by the mean number of people who had undergone sequencing for that gene in gnomAD. This presumed that each affected person had only one pathogenic change in an ADPLD gene. Biallelic variants have been reported in ADPLD [30], and a ‘two hit hypothesis’ with 2 pathogenic variants is consistent with the variability in the number and size of cysts [31]. It is possible but laborious to identify compound heterozygous variants in a single gene in gnomAD and it is not possible currently to identify digenic variants in the same person.

The population frequencies were also calculated from the number of predicted pathogenic variants in the Control subset (n = 60,146) in gnomADv.2.1.1.

### Population frequencies of predicted pathogenic variants in the ADPLD genes in gnomAD v.2.1.1 using ClinVar, LOVD and HGMD

The population frequencies of predicted pathogenic variants in ADPLD genes were also estimated from gnomAD variants assessed as disease-causing in ClinVar (Pathogenic, Likely pathogenic or Conflicting (Variant of Uncertain Significance (VUS) plus Pathogenic or Likely pathogenic, but not Benign or Likely benign)) (https://www.ncbi.nlm.nih.gov/clinvar/) from LOVD (https://databases.lovd.nl/shared/genes); or from HGMD (https://www.hgmd.cf.ac.uk/ac/index.php) even if variants were found in more than 5 people. This approach was taken because assessments were made on sequencing from people tested by accredited diagnostic genetic laboratories, who used the ACMG/AMP criteria [20] and recent population datasets for rarity. While errors occur in database assessments, these have become fewer with time [32], and this strategy enabled us to detect common founder variants.

### Population frequency for predicted pathogenic variants in the ADPLD genes using gnomAD v.4.1

The results from gnomAD v.2.1.1 were then compared with the replication dataset from the ClinVar assessment of variants in gnomAD v.4.1, which included structural and copy number changes.

Variants in individual ADPLD genes were again downloaded from gnomAD v.4.1, and checked for assessments in ClinVar. Variants that were reported as Pathogenic, Likely pathogenic, or Conflicting (VUS plus Pathogenic or Likely pathogenic) were assessed as predicted pathogenic even if they were found in more than 5 people.

The total number of people with a predicted pathogenic variant in gnomAD v.4.1 included those with a loss of function structural or copy number change that affected an exon where numbers from the subsets were corrected to be equivalent to the whole cohort, in addition to the changes assessed as disease-causing in ClinVar.

The population frequency for ADPLD was calculated from the total number of people with a predicted pathogenic variant in our assessment of gnomAD v.4.1 divided by the mean number of people who had undergone WES and WGS for the gene in gnomAD, and, again, presumed that each person had only one pathogenic variant in an ADPLD gene.

### Population frequencies of predicted pathogenic variants in ADPLD genes in people of different ancestries

Population frequencies of predicted pathogenic variants were then derived from gnomAD v.4.1 for people of African/African American, Admixed/American, Ashkenazim, East Asian, Finnish, Middle Eastern, European (Non-Finnish), South Asian or Remaining ancestries. These estimates included structural and copy number variants.

### Statistical analysis

Results were compared with Chi-squared calculations with Yates’ correction using Graphpad (https://www.graphpad.com/).

## Results

### Population frequency of predicted pathogenic variants in the ADPLD genes using gnomAD v.2.1.1

#### Population frequency of predicted pathogenic variants in the ADPLD genes using our strategy.

Overall, 18 of the 20 Benign variants were benign in our assessment, and 15 of the Pathogenic variants from ClinVar were predicted pathogenic. This corresponded to a 75% sensitivity, 90% specificity, and an 88% positive and 78% negative predictive value (S1 Table).

Overall, there were 644 predicted pathogenic variants in 1,268 people in the 7 ADPLD genes (*GANAB*, *ALG8, ALG9, LRP5, PRKCSH, SEC61B,* and *SEC63*) in 115,143 people corresponding to a population frequency of one in 91 for ADPLD (Table 2, Suppl Tables S2-S8 in S1 Data). The commonest affected genes were *LRP5* (one in 274), and *ALG8* (one in 277), and the least common was *SEC61B* (one in 12,146). No pathogenic structural variants were found in *ALG9*, *GANAB*, *LRP5* or *SEC61B*, and predicted pathogenic missense variants were about as common (n = 675) as null (n = 537) changes overall.

**Table 2:** Population frequencies for predicted pathogenic variants in ADPLD genes using our strategy (gnomAD v.2.1.1).

Gene (mean number of people sequenced)	Predicted pathogenic structural variants (corrected for smaller cohort n = 10,847)		Predicted pathogenic null variants		Predicted pathogenic missense variants		Total predicted pathogenic variants		No. of people with a predicted pathogenic variant in mean number of people studied	Population frequency in whole cohort
	No. of variants	No. of people	No. of variants	No. of people	No. of variants	No. of people	No. of variants	No. of people		
***GANAB*** **(n = 120,987)**	0	0	21	26	5	9	26	35	35 in 120,987	One in 3,457
***ALG8*** **(n = 112,484)**	14 (1x14)	28 (2x14)	41	259	76	119	131	406	406 in 112,484	One in 277
***ALG9* (n = 117,325)**	0	0	17	21	20	30	37	51	51 in 117,325	One in 2,300
***LRP5*** **(n = 119,028)**	0	0	45	51	210	384	255	435	435 in 119,028	One in 274
***PRKCSH*** **(n = 115,260)**	14 (1x14)	14 (1x14)	37	126	46	67	97	207	207 in 115,260	One in 557
***SEC61B*** **(n = 109,313)**	0	0	3	3	6	6	9	9	9 in 109,313	One in 12,146
***SEC63*** **(n = 113,786)**	14 (1x14)	14 (1x14)	32	51	43	60	89	125	125 in 113,786	One in 910
**All genes** **(n = 115,143)**	42	56	196	537	406	675	644	1,268	1,268 in 115,143	One in 91

#### Population frequency of predicted pathogenic variants in the ADPLD genes in the control cohort.

There were 548 people with a predicted pathogenic variant in the control cohort of 50,404 people corresponding to a population frequency of ADPLD of one in 92 people, which was not different from our result for the whole gnomAD v.2.1.1 cohort (p = 0.99) (Table 3). However the control cohort did not include structural or copy number changes, which suggested a population frequency that was even more greater.

**Table 3:** Population frequency of predicted pathogenic variants in ADPLD genes using our strategy, controls and other variant databases (gnomADv.2.1.1).

Gene	Our assessment of whole cohort		Our assessment of Controls		ClinVar P/LP and Conflicting (VUS/P/LP)	LOVD Pathogenic or Likely pathogenic	HGMD Pathogenic
	No. people with predicted pathogenic variant	No. of people tested	No. people with predicted pathogenic variant	No. of people tested	No. of predicted pathogenic variants and people with a variant	No. of predicted pathogenic variants and people with a variant	No. of predicted pathogenic variants and people with a variant
***GANAB* (n = 120,987)**	35	120,987	19	52,018	4 in 4 people	1 in 1 person	3 in 12 people
	One in 3,457		One in 2,738		One in 30,247	One in 120,987	One in 10,987
***ALG8*** **(n = 112,484**	406	112,484	187	49,422	10 in 139 people	6 in 51 people	23 in 176 people
	One in 277		One in 264		One in 809	One in 2,206	One in 639
***ALG9 (n = 117,325)***	37	51	24	49,947	4 in 6 people	Not present	Not present
	One in 2,300		One in 2,081		One in 19,554	Not present	Not present
***LRP5*** **(n = 119,028)**	435	119,028	178	51,344	53 in 189 people	13 in 28 people	109 in 2142
	One in 274		One in 288		One in 630	One in 4,251	One in 56
***PRKCSH* (n = 115,260)**	207	115,260	84	50,794	7 in 12 people	1 in 1 person	10 in 704 people
	One in 557		One in 605		One in 9,605	One in 115,260	One in 164
***SEC61B* (n = 109,313)**	9	109,313	5	48,500	None	None	None
	One in 12,146		One in 9,700				
***SEC63*** **(n =** **113,786)**	125	113,786	51	50,350	7 in 12 people	1 in 3 people	7 in 21 people
	One in 910		One in 987		One in 9,482	One in 37,929	One in 5,418
**Total number of variants**	1,268 in 115,143 people		548 in 50,404 people		362 variants in 115,143 people	24 variants in 115,143 people	3,055 variants in 115,143 people
**Total**	One in 91 people Ref		One in 92 people p = 0.99		One in 318 P < 0.0001	One in 5,041 P < 0.0001	One in 40 P < 0.0001

*The Control cohort did not include sructural or copy number variants, and only included missense variants found in fewer than 5 people.

#### Population frequency of predicted pathogenic variants in the ADPLD genes using ClinVar, LOVD or HGMD databases.

Overall, the population frequencies of disease-causing variants in the ADPLD genes in gnomAD v.2.1.1 assessed by other variant databases were one in 318 using ClinVar, one in 40 using HGMD, and one in 5,041 using LOVD (Table 3). No pathogenic variants were found in *SEC61B* using ClinVar, LOVD or HGMD.

### Predicted pathogenic variants in the ADPLD genes using ClinVar in gnomAD4.1

The overall population frequency for ADPLD was one in 130 people according to the ClinVar assessments of variants in these 7 genes (*GANAB*, *ALG8, ALG9, LRP5, PRKCSH, SEC63, SEC61B)* (Table 4). Only copy number variants were found in *SEC61B* in gnomAD v.4.1 using ClinVar. Again null variants (n = 2,736) were about as common as missense changes (n = 2,616).

**Table 4:** Pathogenic variants in ADPLD genes using ClinVar and pathogenic structural and copy number variants (gnomAD v.4.1).

Gene (number of people tested)	Pathogenic Structural variants (x 16)		Pathogenic Copy number variants (x 1.74)		Pathogenic null variants in ClinVar		Pathogenic missense variants in ClinVar		Total Pathogenic variants		No. of people with a predicted pathogenic variant in mean number of people studied	Population frequency in whole cohort
	No. of variants	No. of people	No. of variants	No. of people	No. of variants	No. of people	No. of variants	No. of people	No. of variants	No. of people		
***GANAB*** ***(n = 805,322)***	**0**	**0**	**3 (5)**	**4 (7)**	**12**	**36**	**2**	**6**	**19**	**49**	**49 in 805,322**	**One in 16,435**
***ALG8*** ***(n = 721,959)***	**5 (80)**	**13 (208)**	**5 (9)**	**6 (11)**	**21**	**897**	**5**	**59**	**115**	**1175**	**1,175 in 721,959**	**One in 614**
***ALG9*** ***(n = 790,262)***	**None**		**3 (6)**	**6 (11)**	**5**	**24**	**2**	**802**	**7**	**826**	**13 in 837 people**	**One in 944**
***LRP5 (n = 789,511)***	**5 (80)**	**21 (336)**	**4 (7)**	**5 (9)**	**31**	**350**	**65**	**1745**	**183**	**2440**	**2,440 in 789,511**	**One in 324**
***PRKCSH (n = 798,572)***	**2 (32)**	**2 (32)**	**3 (5)**	**3 (6)**	**16**	**290**	**2**	**2**	**23**	**330**	**330 in 798,572**	**One in 2,420**
***SEC61B***	**0**	**0**	**4**	**4**	**0**	**0**	**0**	**0**	**0**	**0**	**0**	**0**
***SEC63*** ***(n = 796,472)***	**2 (32)**	**2 (32)**	**4 (7)**	**4 (7)**	**14**	**1139**	**1**	**2**	**54**	**1180**	**1,180 in 796,472**	**One in 674**
**Total** **(n = 782,367)**	**224**	**608**	**43**	**55**	**99**	**2,736**	**77**	**2,616**	**401**	**6,000**	**6,000 781,274**	**One in 130**

Again the commonest predicted pathogenic variants in the ADPLD genes were *LRP5* (one in 324) and *ALG8* (one in 614). *SEC63* variants were also common (one in 674).

#### Population frequencies of predicted pathogenic variants in ADPLD genes in gnomADv.4.1 in different ancestries.

ADPLD appeared to be commonest in African/African-Americans (one in 43) and Admixed Americans (one in 91), compared with Europeans (one in 187, p < 0.0001) (Table 5).

**Table 5:** Population frequencies of predicted pathogenic variants in ADPLD genes in people of different ancestries (gnomAD.v4.1).

Gene	African/African American	Admixed American	Ashkenazim	East Asian	Finnish	Middle Eastern	European (non-Finnish)	South Asian	Remaining
***GANAB***	Total 37,463 (n = 1) One in 37,463 P = 0.40	Total 29,979 (n = 1) One in 29,979 P = 0.55	Total 14,786 (n = 1) One in 14,786 P = 0.92	Total 22,421 (n = 0)	Total 31,995 (n = 0)	Total 3,015 (n = 1) One in 3,015 P = 0.06	Total 588,534 (n = 36) One in 16,366 Ref	Total 45,488 (n = 2) One in 22,744 P = 0.65	Total 31,186 (n = 2) One in 15,593 P = 0.95
***ALG8***	Total 35,453 (n = 118) One in 300 P < 0.0001	Total 27,369 (n = 60) One in 456 P < 0.0001	Total 13,427 (n = 1) One in13427 P = 0.0001	Total 20,126 (n = 25) One in 805 P = 0.96	Total 28,899 (n = 182) One in 159 P < 0.0001	Total 2,742 (n = 2) One in 1371 P = 0.46	Total 524,443 (n = 644) One in 814 Ref	Total 41,241 (n = 5) One in 8248 P < 0.0001	Total 27,804 (n = 47) One in 595 P = 0.04
***ALG9***	Total 37,392 (n = 591) One in 63 p < 0.0001	Total 27,340 (n = 28) One in1,038 p < 0.0001	Total 14,566 (n = 1) One in 14,566 P = 0.058	Total 21,853 (n = 1) One in 21,853 P = 0.05	None	Total 2,413 (n = 4) One in741 P = 0.0003	Total 589,969 (n = 152) One in 3,805 Ref	Total 43,529 (n = 5) One in 8,941 P = 0.001	Total 31,681 (n = 44) One in 646 P < 0.0001
***LRP5***	Total 36,953 (n = 136) One in 272 P = < 0.0001	Total 28,907 (n = 140) One in 206 P < 0.0001	Total 14,266 (n = 10) One in 1465 P = 0.0004	Total 21,378 (n = 29) One in 737 P = 0.03	Total 29,883 (n = 19) One in 1573 P < 0.0001	Total 2,921 (n = 2) One in 1461 P = 0.11	Total 580,307 (n = 1177) One in 493 Ref	Total 44,098 (n = 110) One in 401 P = 0.04	Total 30,344 (n = 46) One in 660 P = 0.05
***PRKCSH***	Total 37,226 (n = 6) One in 6204 P = 0.008	Total 29,361 (n = 19) One in 1545 P = 0.14	Total 14,647 (n = 0)	Total 22,204 (n = 1) One in 22204 P = 0.004	Total 31,450 (n = 34) One in 925 P < 0.0001	Total 2,963 (n = 0)	Total 584,431 (n = 266) One in 2195 Ref	Total 44,905 (n = 0)	Total 3,0931 (n = 3) One in 10310 P = 0.003
***SEC61B***	(n = 0)	(n = 0)	(n = 0)	(n = 0)	(n = 0)	(n = 0)	(n = 0)	(n = 0)	(n = 0)
***SEC63***	Total 36,838 (n = 27) One in 1364 P = 0.0002	Total 29,474 (n = 81) One in 365 P < 0.0001	Total 14,675	Total 22,238	Total 31,608	Total 2,953	Total 582,351	Total 44,993	Total 30,893
			(n = 26) One in 570 P = 0.42	(n = 15) One in 1483 P = 0.002	(n = 56) One in 567 P = 0.23	(n = 1) One in 2953 P = 0.10	(n = 871) One in 669 Ref	(n = 65) One in 692 P = 0.79	(n = 39) One in 792 P = 0.30
**Total in ClinVar**	Total 37,463 (n = 872) One in 43 P < 0.0001	Total 29,979 (n = 329) One in 91 P < 0.0001	Total 14,786 (n = 39) One in 379 P < 0.0001	Total 22,421 (n = 71) One in 316 P < 0.0001	Total 31,995 (n = 291) One in 110 P < 0.0001	Total 3,015 (n = 10) One in 302 P < 0.0001	Total 588,534 (n = 3,146) One in 187 Ref	Total 45,488 (n = 187) One in 243 P = 0.002	Total 31,186 (n = 181) One in 172 P = 0.32

## Discussion

Our assessment found that the population frequency of predicted pathogenic variants for the ADPLD genes (*GANAB, ALG8, ALG9, LRP5, PRKCSH, SEC61B, SEC63*) was one in 91 using our assessment for the gnomADv.2.1.1 cohort, and one in 130 using ClinVar assessments of gnomADv.4.1. The difference is explained by the inclusion of structural and copy number changes in gnomAD4.1 but also the lack of ClinVar assessments for all gnomAD v.4.1 changes.

However genetic causes of liver cysts are probably even more common. Up to 80% of people with typical ADPKD have liver cysts [33] and we have used a similar strategy to demonstrate that typical ADPKD (*PKD1*, *PKD2* variants) affects one in 314 people, which corresponds to liver cysts from these genes occurring in one in 378 people. This means that, overall, liver cysts from pathogenic variants in the ADPKD and ADPLD genes occur in at least one in 73 of the population. There are even more genetic causes of liver cysts such as the atypical forms of ADPKD (*ALG9, DNAJB11, ALG5, IFT140, NEK8*) that were not considered here and further still-to-be-identified genes.

There are also other non-genetic reasons why our population frequencies may be underestimates. gnomAD v.2.1.1 does not include data for structural and copy number changes in everyone, and our strategy was relatively insensitive for pathogenic missense changes (75%), and will have excluded founder variants because of the requirement that the missense allele frequency was ≤ 5.

Nevertheless not everyone with a predicted pathogenic variant in an ADPLD-associated gene develops liver cysts nor indeed kidney cysts because of incomplete penetrance and variable expressivity [19]. Missense variants which are typically associated with less penetrant and milder disease were common in all these genes except for *SEC61B*.

Assessments of both gnomAD v.2.1.1 and v.4.1 demonstrated that the commonest genes affected in polycystic liver disease were *LRP5* and *ALG8. SEC1B* variants were rare, and their role in liver cyst development ‘limited’ according to the ClinVar gene-disease association framework. Indeed, ClinVar also considers the evidence for *LRP* in ADPLD to be limited. These results differ from earlier reports that suggested *PRKCSH* and *SEC63* variants were the commonest disease-causing genes, accounting for up to one third of cases of cystic liver disease [16,34], and that *LRP5* variants accounted for fewer than 1% [35]. However the early studies were based on small cohorts of patients with severe disease, while our population frequencies were derived from all pathogenic variants in large cohorts of normal people including some likely without liver cysts.

Most of the ADPLD genes considered here are also associated with kidney cysts. Pathogenic variants in *ALG8, ALG9* and *GANAB* are commonly associated with kidney cysts, but variants in *SEC63* less so. Variants in *LRP5* and *SEC61B* may not be associated with kidney cysts at all. The number of ADPLD-associated kidney cysts is very variable and kidney failure is uncommon [36]. We have previously demonstrated that predicted pathogenic variants in the atypical ADPKD genes affect about one in 283 people. Since most predicted pathogenic variants in ADPLD genes are also associated with kidney cysts this means that the population frequency of polycystic kidney disease is even more common than one in 283.

The strengths of this study were the use of two assessment strategies that included our approach, and confirmation using variant databases, especially ClinVar, in a minimally-overlapping gnomAD dataset. Our approach’s main advantage was that it examined each variant in gnomAD v.2.1.1 using the ACMG/AMP criteria based on rarity and computationally-assessed pathogenicity. However individual variant assessments were less accurate because of the limitations of the computational tools, the lack of confirmatory clinical data, and because common founder variants may have been excluded.

On the other hand, individual variant assessments of pathogenicity with ClinVar are mostly accurate with 84% concordance in the assessments between laboratories [37] and improving over time [32] because they have been submitted from accredited genetic testing laboratories, and use ACMG/AMP criteria and rarity based on large datasets. For these reasons, all variants considered pathogenic by ClinVar were used to calculate the population frequencies in gnomADv.4.1, no matter how often they were found. However, ClinVar included no pathogenic *SEC61B* variants at all [38]. Importantly, ClinVar does not assess each gnomAD variant meaning that the corresponding population frequencies represent underestimates. Population frequencies derived from variants characterised as pathogenic in LOVD or HGMD share some of the limitations of the ClinVar assessments. They may also include more errors because many of their variants were reported prior to the availability of the ACMG/AMP criteria and of the large variant datasets used to evaluate rarity [37].

The study’s major limitations were the lack of clinical confirmation of variant pathogenicity, underestimation of structural and copy number changes, and errors, especially in our interpretation of missense changes.

On the other hand, the alternative strategy included all disease-causing variants found in ClinVar, LOVD or HGMD even when they were very common which may have inflated the corresponding population frequencies. ClinVar variants can still be inaccurate. These inaccuracies arise from outdated classifications, improved ACMG guidelines and subjective interpretations from different clinical testing laboratories.

Our strategy also required missense variants to be positive in all three computational tools (PP2, SIFT, Mutation Taster) rather than using a meta score such as REVEL or BAYESdel. Both REVEL and BAYESdel incorporates the same three tools that we used but the advantage of the better tools may be diminished by the less well- performing ones. In addition, our population frequencies assumed that each person had only one pathogenic variant in one gene but some people possibly had two disease-causing variants, even in different genes [30]. The two-hit hypothesis of cyst formation might explain the variation in penetrance and variability in clinical presentation [31].

We have estimated the population frequencies of predicted pathogenic variants as a surrogate for ADPLD but are uncertain of how often cystic liver disease results, because the penetrance of individual genes and, indeed, of types of variants in the ADPLD genes is not known. In general, the number of liver cysts and the number of kidney cysts are highly variable.

Two major efforts will improve the accuracy of these estimates of the prevalence of ADPLD and of associated kidney cysts. One is the use of biobanks that include both genomic variants and imaging data. The other is a systematic classification of all the variants in the ADPLD genes in gnomAD using the ACMG/AMP criteria and all available evidence including previously reported cases. However this will still include errors. Our approach and the use of ClinVar variants was less laborious but not flawless.

In conclusion, this study demonstrated that predicted pathogenic variants in the ADPLD genes are more common than suspected previously. These population frequencies will become more accurate as computational tools improve, and more ADPLD genes and founder variants identified. Variants in ADPLD genes may be reported when genetic testing for kidney cysts is performed or a broad genetic kidney panel is examined, and it is useful for renal physicians to be familiar with these genes, and their contribution to the kidney cysts seen in their patients.

## Supporting information

S1 Data Suppl data ADPLD. (XLSX)
